# Structural Characterization of Canine Minute Virus, Rat and Porcine Bocavirus

**DOI:** 10.3390/v15091799

**Published:** 2023-08-24

**Authors:** Michael Velez, Mario Mietzsch, Jane Hsi, Logan Bell, Paul Chipman, Xiaofeng Fu, Robert McKenna

**Affiliations:** 1 Department of Biochemistry & Molecular Biology, University of Florida, Gainesville, FL 32610, USA; 2 Biological Science Imaging Resource, Department of Biological Sciences, Florida State University, Tallahassee, FL 32306, USA

**Keywords:** parvovirus, bocavirus, capsid, cryo-EM, pathogen, CnMV, PBoV, RBoV

## Abstract

*Bocaparvovirus* is an expansive genus of the *Parvovirinae*, with a wide range of vertebrate hosts. This study investigates Canine minute virus (CnMV), Rat bocavirus (RBoV), and Porcine bocavirus 1 (PBoV1). Both CnMV and PBoV1 have been found in gastrointestinal infections in their respective hosts, with CnMV responsible for spontaneous abortions in dogs, while PBoV has been associated with encephalomyelitis in piglets. The pathogenicity of the recently identified RBoV is currently unknown. To initiate the characterization of these viruses, their capsids structures were determined by cryo-electron microscopy at resolutions ranging from 2.3 to 2.7 Å. Compared to other parvoviruses, the CnMV, PBoV1, and RBoV capsids showed conserved features, such as the channel at the fivefold symmetry axis. However, major differences were observed at the two- and threefold axes. While CnMV displays prominent threefold protrusions, the same region is more recessed in PBoV1 and RBoV. Furthermore, the typical twofold axis depression of parvoviral capsids is absent in CnMV or very small in PBoV and RBoV. These capsid structures extend the structural portfolio for the *Bocaparvovirus* genus and will allow future characterization of these pathogens on a molecular level. This is important, as no antivirals or vaccines exist for these viruses.

## 1. Introduction

*Parvoviridae* is a family of small, non-enveloped viruses with a linear single-stranded (ss) DNA genome of around 4–6 kb [1]. The family is divided into three subfamilies, *Parvovirinae*, *Densovirinae*, and *Hamaparvovirinae*. Members of the *Parvovirinae* exclusively infect vertebrates, *Densovirinae* infect invertebrates, whereas the *Hamaparvovirinae* subfamily contains members that infect either vertebrates or invertebrates [2]. Within the *Parvovirinae* subfamily, the *Bocaparvovirus* genus is the largest, with currently 31 described member species, containing a large number of pathogenic viruses [3].

Bocaviruses possess a ~5.5 kb ssDNA genome with two flanking terminal repeats at either end, involved in replication and genome packaging [4]. Located between these repeats are three major open reading frames (ORFs), NS1, NP1, VP, and a single promoter. Unique to bocaviruses is the NP1 ORF located between the NS1 and VP ORF [5], which was shown to play a role in mRNA processing and subsequent capsid protein expression [5]. The NS1 ORF contains the *ns* gene coding for non-structural protein 1 (NS1), which is responsible for replication and DNA packaging [6]. The VP ORF contains the *cap* gene, which expresses the structural or viral proteins VP1 and VP2 (in addition, some bocaviruses also express a third, VP3) [1,7]. The VPs overlap at their C-termini, with VP2 completely contained in VP1. VP1 possesses a unique N-terminal region (VP1u) with an enzymatic phospholipase A_2_ motif, needed for endosomal/lysosomal escape during infection [8], and is expressed approximately tenfold less compared to VP2. Thus, the assembled T = 1 icosahedral capsid, consisting of 60 VPs, is primarily composed of VP2 [7,9].

To date, the capsid structures of six bocaviruses have been determined, either by X-ray crystallography in the case of bovine parvovirus (BPV) [10] or by cryo-electron microscopy (cryo-EM) in the case of the human bocaviruses 1–4 (HBoV1-4) and gorilla bocavirus 1 (GBoV1) [11,12,13]. Their VP structures contain a conserved eight-stranded jelly roll motif (βB-βI), an additional beta-strand antiparallel to βB, and two alpha helices. The connecting loops between the β-strands are named based on the flanking beta-strands (e.g., DE-loop). These loops form the capsid surface of the virus and display the highest sequence and structural variability within the genus and to other parvoviruses. Ten variable regions (VRs) have been defined for the bocaviruses [14]. In the icosahedral capsid, the 60 VPs assemble via two-, three-, and fivefold symmetry-related VP interactions, resulting in pores at the fivefold symmetry axes, protrusions surrounding the threefold axes, and depressions at the twofold axes [9].

Canine Minute Virus (CnMV) was first isolated from German military dogs in 1967 and was initially thought to be non-pathogenic [15]. Later, it was found to be pathogenic in newborn puppies and fetuses, as the virus was found in the lungs and intestines of nursing puppies, leading to lesions, diarrhea, and death [16]. The CnMV infections were also found to be responsible for abortions of fetuses in dogs [17]. Furthermore, the virus has recently been indicated as a cause for hepatitis in dogs [18]. The capsid (VP1) amino acid sequence identity is 40% and 45% to BPV and HBoV1, respectively.

The first porcine bocavirus (PBoV) was identified in Swedish pigs in 2009 [19]. It was found to be a co-infective agent with Porcine circovirus 2 (PCV2) in postweaning multisystemic wasting syndrome. It has since then been identified in pigs globally, disproportionally affecting piglets aged 3–6 months [20]. It is often found in co-infections of piglets with PCV2, Porcine Torque teno virus (PTTV), Porcine reproductive and respiratory syndrome virus (PRRSV), Classical swine fever virus (CSFV), Porcine epidemic diarrhea virus (PEDV), Porcine kobuvirus (PKoV), Group A rotavirus (GARV), and Transmissible gastroenteritis virus (TGEV) [21,22,23,24]. PBoV is usually associated with diarrhea and upper respiratory tract infections in piglets, but a case study in 2016 associated PBoV with a case of encephalomyelitis in a 6-week-old piglet in Germany [24]. Noteworthy, PBoV infections are not exclusive to pigs. In 2018, PBoV was isolated from an upper respiratory infection in a 3-year-old child in northeastern Iran [25], raising public health concerns, considering the fact that these animals are found in close proximity with humans, and these viruses have the capacity to jump to a human host. There are a large number of PBoV strains, which exhibit significant genetic variability [26]. The capsid (VP1) amino acid sequence identity of PBoV1 is 42% and 47% for BPV and HBoV1, respectively.

Rodents are a large and diverse order of mammals living in close proximity to humans, making them an optimal reservoir for emerging pathogens [27]. This characteristic makes identifying pathogens in rodent populations a necessary precaution for public health. In 2017, rat bocavirus (RBoV) was identified from brown rats in China. As a recent addition to the *Bocaparvovirus* genus, its pathogenicity is still unknown, but has been shown to have broad tissue tropism [28]. Its capsid (VP1) amino acid sequence identity is 37% and 41% for BPV and HBoV1, respectively.

The goal of this study was to structurally characterize the capsids of CnMV, PBoV1, and RBoV to obtain further insights into the structural repertoire of this genus of the *Parvovirinae*. The high-resolution structures for these virus capsids, determined using cryo-electron microscopy (cryo-EM) and 3D image reconstruction, are reported. The capsids shared features characteristic of all parvoviruses, such as the fivefold channel and threefold protrusion, but CnMV and RBoV diverge from the other bocaviruses, as they lack a twofold depression. The VP2 monomer of all three viruses exhibited the conserved features observed in other major capsid proteins within the genus, such as the 8-stranded beta-sheet, but also varied, with an additional alpha helix (αC) observed. High variance was observed in VR III and IX, which appeared to translate to the lack of the twofold depression in the CnMV and RBoV capsid. Additionally, insertions in VR IV, V, and VIII are responsible for a change in the phenotype of the threefold protrusions for these viruses. These observations help establish a structural platform for further research into these viruses and the *Bocaparvovirus* genus.

## 2. Materials and Methods

### 2.1. Virus-like Production and Purification

Virus-like particles (VLPs) of CnMV, PBoV, and RBoV were expressed using the Bac-to-Bac baculovirus system, as described previously and following the manufacturer’s protocol [29]. The open reading frames for VP2 were synthesized (Azenta/Genewiz) and inserted into the pFastBac plasmid (CnMV: nt 3329-5044, accession # NC_004442.1; PBoV1: nt 3417-5120, accession # NC_024453.2; RBoV: nt 32624965, accession # NC_029133.1). For VLP production, suspension Sf9 insect cells were cultured in Sf-900 II SFM media and infected with the recombinant baculoviruses at a multiplicity of infection of 5 plaque-forming units. The cells were harvested 72 h post infection and pelleted by centrifugation at 1000× *g* for 20 min at 4 °C. The Sf9 cell pellets were resuspended in TNTM buffer (25 mM Tris-HCl, 100 mM NaCl, 0.2% Triton X-100, 2 mM MgCl_2_, pH 8.0) and subjected to three freeze–thaw cycles. The lysates were benzonase-treated (125 U/mL) for 1 h at 37 °C and clarified by centrifugation at 12,000× *g* for 30 min to remove cell debris. The supernatant was loaded onto a 20% sucrose cushion (*w*/*v* sucrose in TNTM buffer) and centrifuged at 45,000 rpm, using a Ti70 rotor for 3 h at 4 °C. The resulting pellet was resuspended in TNTM buffer, loaded onto a 10–40% sucrose step gradient (*w*/*v* sucrose in TNTM buffer), and centrifuged using a SW41 rotor for 3 h at 4 °C. Individual fractions were recovered from the gradient and analyzed by SDS-PAGE. Fractions containing the expected VP2 band at ~60 kDa were dialyzed in phosphate-buffered saline (PBS), concentrated to > 0.5 mg/mL using Apollo concentrators (Orbital Biosciences, Topsfield, MA, USA), and stored at −20 °C. The integrity of the capsids was analyzed via negative stain electron microscopy using a Tecnai G2 Spirit electron microscope at 200 kV, as described previously [30].

### 2.2. Cryo-EM Data Collection

Aliquots of the purified CnMV, PBoV1, and RBoV samples were applied to glow discharged, holey carbon grids and the grids vitrified in liquid ethane using a Vitrobot Mark 4 (FEI) at 95% humidity and 4 °C. The grids were screened for the VLP’s particle distribution using a Tecnai G2 F20-TWIN transmission electron microscope (FEI) at 200 kV under low-dose conditions (20 e^−^/Å^2^) at a magnification of 82,500-fold on a 16-megapixel charge-coupled device (CCD) camera. High-resolution data for the CnMV sample were collected at the Florida State University through the Southeastern Consortium for Microscopy of Macro Molecular Machines (SECM4), using a Titan Krios electron microscope with a DE-64 detector. The microscope was run at 300 kV, with a total dose of 59 e^−^/Å^2^ to collect 52 frames per micrograph. Data for RBoV and PBoV were collected at the Stanford-SLAC Cryo-EM Center (S^2^C^2^), using a Titan Krios (FEI) electron microscope operated at 300 kV, equipped with a Falcon 4 direct electron detector (Thermo Fisher). A total of 50 movie frames were collected per micrograph at a total electron dose of ~50 e^−^/Å^2^. The movie frames were aligned using MotionCor2, as described previously [31].

### 2.3. Three-Dimensional Particle Reconstruction

The software package cisTEM was used to reconstruct the individual two-dimensional images to three-dimensional electron density maps of the reported viruses, as reported previously [32]. Briefly, the aligned micrographs were imported and their contrast transfer functions (CTFs) calculated. Micrographs of poor quality were removed and capsids automatically picked using a characteristic particle radius of 125 Å. The individual capsid images were sorted via 2D classification into 20 classes. Classes containing impurities were discarded. The Ab-Initio 3D function was utilized to generate an initial map from 10% of the particles. This map was further refined using the automatic refinement function with default settings. The resolutions of the reported maps were determined at a Fourier shell correlation (FSC) criterion threshold of 0.143. The final electron density maps were sharpened using the pre-cut off B-factor value of −90 Å^2^ and variable post-cut off B-factor values of 0, 20, and 50 Å^2^.

### 2.4. Model Building and Structure Refinement

Initial atomic VP models for CnMV, RBoV, and PBoV1 were generated with SWISS-MODEL, using their primary amino acid sequences and the capsid structure of HBoV1 as a template [33]. Full capsid models (60 copies of VP2) were created using VIPERdb2 oligomer generator [34]. These models were fitted in their respective density map in UCSF-Chimera [35] and their pixel size adjusted using MAPMAN [36]. The EM density maps and monomer models of the viruses were then imported into Coot, where manual model building and real-space refinement tools were used to improve the capsid models to better fit their electron density maps [37]. Finally, the 60-mer capsid models were automatically refined, using the real-space-refine subroutine in PHENIX with default settings, which also provided refinement statistics [38] (Table 1).

### 2.5. Structural Comparison

The capsid surface morphology of CnMV, RBoV, PBoV, and HBoV1 were visually compared using Chimera, while the VP2 models of these viruses were superposed in Coot to obtain overall paired RMSDs between Cα positions and to identify regions of structural similarities and differences. Deviations between non-overlapping Cα positions, because of residue deletion/insertions, were measured using the distance tool in Coot. Regions of two or more adjacent amino acids with ≥2.0 Å difference in superposed VP2 Cα position were considered to be structurally diverse and assigned to the previously described VRs. This information was also used for a structure-based sequence alignment, and to calculate the structural identity (in %) that was defined as the number of aligned residues (≤2.0 Å apart) divided by the total number of residues. Amino acid sequence alignments of the different bocaparvoviruses were performed utilizing the sequence alignment option in VectorNTI (Invitrogen, Waltham, MA, USA).

## 3. Results and Discussion

### 3.1. Expression and Purification of VLPs of CnMV, PBoV, and RBoV

**Figure 1:**
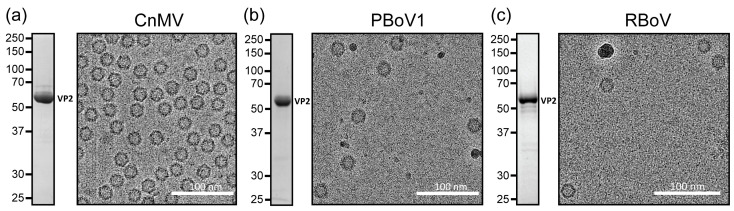
Expression and purification of CnMV, RBoV, and PBoV VLPs. (**a**) An SDS-PAGE of CnMV showing a band at 60 kDa consistent with VP2. To the right, a cryo-EM micrograph of the same sample shows intact capsids of ~25 nm in diameter. (**b**) Depiction as in (**a**) for PBoV1 and for (**c**) RBoV.

To expand the structural repertoire of determined capsid structures for the diverse Bocaparvovirus genus, viruses infecting dogs (CnMV), pigs (PBoV1), and rats (RBoV) were selected. The ORFs of these viruses coding for the major capsid protein VP2 were cloned into the pFastBac1 plasmid to enable the production of the virus-like particles (VLPs), using the Bac-to-Bac expression system in insect Sf9 cells. Following the production and purification of the VLPs, the samples were analyzed for their purity by SDS-PAGE. For all of the three viruses, a band consistent with VP2, migrating at ~60 kDa, was observed (Figure 1). Further analysis of these samples by cryo-EM showed intact capsids of approximately 25 nm in diameter, and thus, they were deemed suitable for high-resolution cryo-EM data collection.

### 3.2. Determination of the CnMV, PBoV1, and RBoV Capsid Structures

**Figure 2:**
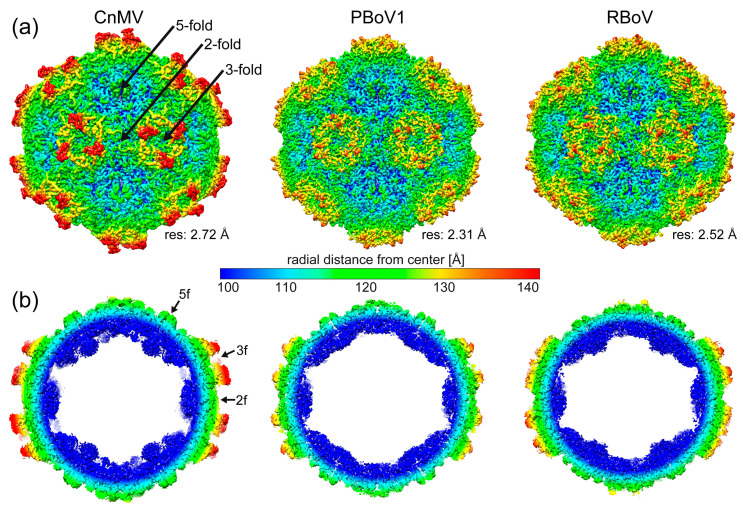
Determination of the CnMV, PBoV1, and RBoV capsid structures. (**a**) Capsid surface density maps of CnMV, PBoV1, and RBoV contoured at a 2 sigma (σ) threshold level. The maps are radially colored (blue to red) according to distance to the capsid center, as indicated by the scale bar in the center. The approximate icosahedral two-, three-, and fivefold axes are indicated. (**b**) Cross-sectional views of the CnMV, PBoV1, and RBoV density map at a 0.5σ threshold.

Following cryo-EM data collection, three-dimensional-image reconstruction of the capsids, utilizing 50,866 (CnMV), 98,388 (PBoV1), and 9199 (RBoV) individual capsid images, resulted in a resolution of 2.72, 2.31, and 2.52 Å, respectively (Table 1). The reconstructed capsids all exhibited a conserved channel at the fivefold symmetry axes surrounded by a depression (Figure 2a). This feature is found in all capsids of the *Bocaparvovirus* genus, as well as other genera of the subfamily determined [9,32]. In contrast, significant differences among these viruses were observed at the two- and threefold regions to each other and to other bocavirus capsids. A depression at the icosahedral twofold axis is a common feature of capsids in the Parvovirinae subfamily. However, for the viruses characterized in this study, the twofold depressions are either insignificant (PBoV1) or non-existent (CnMV). At the threefold axes, typically protrusions are found for the *Parvovirinae*. Capsid structures previously determined for the *Bocaparvovirus* genus showed disperse protrusions surrounding the threefold axis that do not significantly extend radially from the capsid surface [10,11,12,13]. In contrast, the CnMV capsid shows very prominent threefold protrusions, while PBoV1 and RBoV follow the previously described characteristics of the genus (Figure 2a). These differences also result in the CnMV capsid having a larger max. diameter of 289 Å, compared to RBoV1 and RBoV with 275 and 277 Å, respectively. Another feature previously observed for the bocaviruses, with the exception of GBoV1, was the presence of density extending into the interior of the capsid below the fivefold channel [10,11,12,13]. This density was also seen for CnMV, but is absent in the PBoV1 and RBoV capsids (Figure 2b). The region below the fivefold channel is also the location of the N-termini of the ordered VP structure for all parvoviruses. It is characterized by a glycine-rich sequence that is hypothesized to act as a hinge for the externalization of the VP1u to utilize its PLA2 activity [39,40]. In CnMV 16, in PBoV1 20, and in RBoV 19, glycines are found within the first ~40 amino acids. However, the number of glycines does not correlate with the presence of the density below the fivefold channel, as BPV, HBoV1-4, and GBoV1 possess only 10–13 glycines in the corresponding region.

### 3.3. CnMV, PBoV1, and RBoV Possess an Additional Surface α-Helix

**Figure 3:**
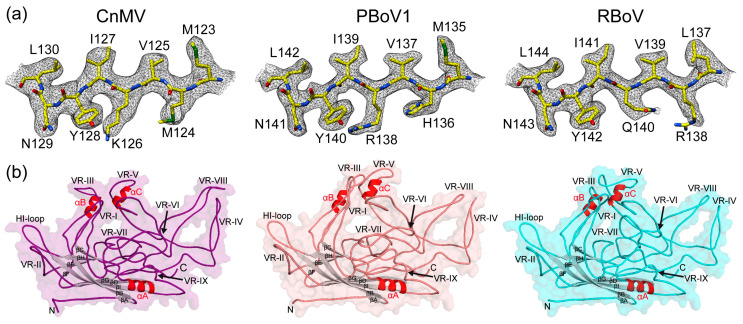
The VP structure of CnMV, PBoV1, and RBoV. (**a**) Amino acid residues modeled for the βD strand are shown inside their density maps at a sigma (σ) threshold of 2.5 (in black). The amino acid residues are labeled and shown as stick representations and colored according to atom type: C = yellow, O = red, N = blue, S = green. These images were generated using UCSF-Chimera [35]. (**b**) The VP structures are shown as ribbon diagrams inside transparent surface representations. The secondary structure elements (α-helices in red, β-strands in gray), the N- and C-termini, and variable regions (VRs) are labeled.

The high resolution of the cryo-EM maps allowed the building of reliable atomic models, starting from amino acids 34, 41, and 42 (VP2 numbering) at the N-terminus for CnMV, PBoV1, and RBoV, respectively. Structural order was observed at the C-terminus with highly ordered amino acid side-chain densities (Figure 3a). In the case of PBoV1 and RBoV, the cryo-EM maps for the most part also showed densities for the carbonyl groups of the main-chain and densities for a specific rotamer for the individual amino acids, guiding the model building for these viruses. The overall models showed good refinement statistics and high map correlation coefficients (CC) of 0.84 to 0.88 (Table 1).

The VP structures of CnMV, PBoV1, and RBoV conserve the features previously described for other bocaviruses, which comprise the eight-stranded jelly roll motif (βB-βI), including the additional beta-strand A and two alpha helices (αA and αB). While the α-helix A (10 amino acids) is part of the core capsid and found in all parvoviruses, α-helix B (8 amino acids) is located at the surface in the VR-III-loop and specific for the *Bocaparvovirus* genus. In the structures described here, an additional α-helix C (seven amino acids) is present in the VR-V loop (Figure 3b). Currently, the only other virus of the *Parvovirinae* with an α-helix at the capsid surface is the Aleutian Mink Disease Virus (AMDV) of the *Amdoparvovirus* genus [32]. However, unlike for the bocaviruses, AMDV’s α-helix is located in the VR-VIII loop.

### 3.4. Structural Differences among the Bocaviruses Are Localized to the Variable Regions

**Figure 4:**
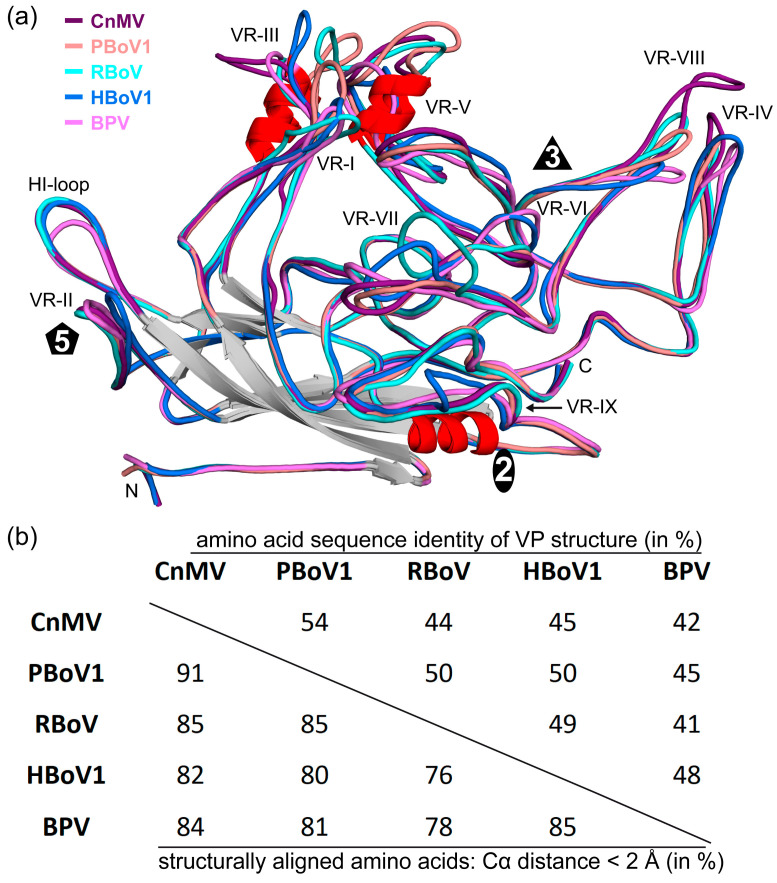
Sequence and structure comparison within the *Bocaparvovirus* genus. (**a**) Superposition of CnMV (purple), PBoV1 (salmon), RBoV (cyan), HBoV1 (blue), and BPV (pink). The variable regions, the N- and C-termini, and the approximate location of the icosahedral two-, three-, and fivefold axes are shown. (**b**) Amino acid sequence identity comparison of the structurally ordered VP region for the given viruses (top right, in %). The structural similarity is shown in the bottom left corner and was defined as the percentage of aligned Cα atoms of the amino acid chain within 2 Å distance when the capsid structures were superposed.

In order to obtain an overview of the structural repertoire within the *Bocaparvovirus* genus, CnMV, PBoV1, and RBoV were compared to each other, but also to previously determined capsids structures of BPV and HBoV1 (Figure 4a). The amino acid sequence identity among these viruses for the structural ordered VP ranges from 41 to 54% (Figure 4b). HBoV1 acts as representative for the other HBoV genotypes and GBoV1 since their sequence identities are in the 77–90% range [12]. As expected, the capsid cores for all bocaviruses are nearly perfectly superposable (Figure 4a). It is also the region where the highest level of sequence conservation is found. In contrast, low sequence identities and significant structural differences were observed in the previously defined VRs for this genus when comparing these five viruses, resulting in overall structural similarities of 76–91% [10]. The least variability is found in the fivefold region with VR-II and the HI-loop (also known as VR-VIIIB). In VR-II, none of the viruses possess an insertion or deletion, and only HBoV1′s loop shows some structural divergence, with a max. Cα-Cα distance of ~6 Å compared to CnMV (Figure 4a). The HI-loop is structurally conserved among the bocaviruses, with the exception of BPV due to a 2 aa deletion in this virus compared to the other bocaviruses. The greater structural conservation of the fivefold region among viruses of the same genus is also observed for the *Dependoparvovirus* and *Protoparvovirus* genera [41,42], likely because the Rep or NS proteins, which usually show higher conservation compared to the capsid, bind to this region during genome packaging [39]. For viruses of different genera, the fivefold regions show significant structural variability [32], possibly reflecting differences between the NS or Rep proteins.

**Figure 5:**
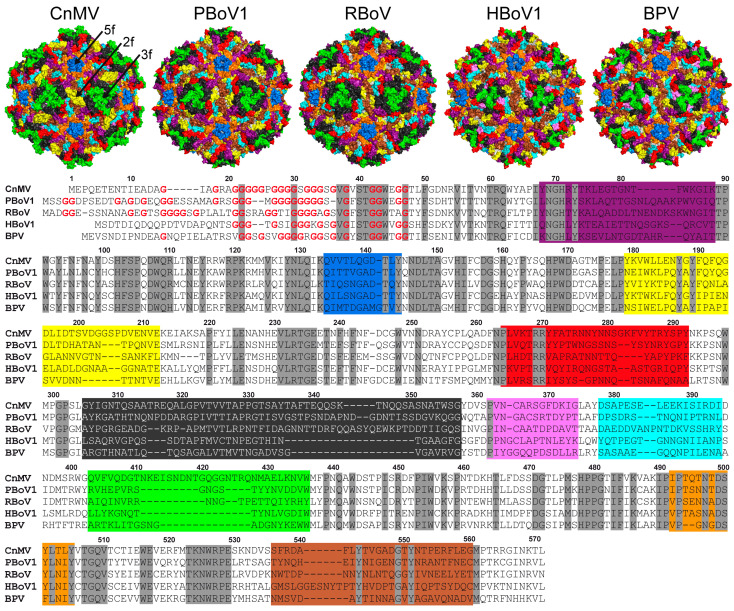
Location of the VRs on the capsid surface. Capsid surface representations of the bocaviruses with the VRs colored (VR-I = purple, VR-II = blue, VR-III = yellow, VR-IV = red, VR-V = dark gray, VR-VI = pink, VR-VII = cyan, VR-VIII = green, HI-loop = orange, VR-IX = brown). The approximate icosahedral two- (2f), three- (3f), and fivefold (5f) axes are indicated on the CnMV capsid. These images were generated using PyMol [47]. The residue range of the colored VRs is shown in the amino acid sequence alignment below. Amino acid numbering, based on the CnMV sequence, is shown above the alignment. Conserved amino acids are highlighted in gray and the glycines near the N-terminus colored red.

The other capsid surface loops show much higher structural variability. The threefold region is primarily composed of VR-IV, -V, and -VIII (Figure 5). The more prominent threefold protrusions of CnMV are the result of significant insertions into VR-IV (+1 aa vs. BPV, +2 aa vs. PBoV1 and HBoV1, +4 aa vs. RBoV) and VR-VIII (+12 aa vs. PBoV1, +13 aa vs. RBoV, +15 aa vs. BPV, +17 aa vs. HBoV1) (Figure 4). Between the threefold protrusions, CnMV’s VR-V is considerably more extensive in all of the newly determined bocaviruses (−3 aa vs. RBoV, −2 aa vs. PBoV1, +17 aa vs. BPV, +18 aa vs. HBoV1). Thus, the presence of α-helix C exclusively in CnMV, PBoV1, and RBoV (Figure 3b) is the result of the additional amino acids in this loop. The threefold region has been shown to display epitopes for neutralizing antibodies and receptor binding sites in other parvoviruses [43,44]. Currently, O-linked α2-3 sialic acid attached to glycophorin A is the only described receptor for BPV in the genus *Bocaparvovirus* [45,46]. However, its binding site has not been determined yet.

The twofold symmetry axis is surrounded by VR-I, VR-III, VR-VI, VR-VII, and VR-IX. Usually, VR-I, VR-III, and parts of VR-IX form the two–fivefold wall on the capsid [10,11]. However, due to the lack of a significant depression at the twofold axis, this capsid feature is absent in the newly determined bocavirus capsids. The lack of any twofold depression in CnMV is caused by an insertion in VR-III (+2 aa vs. HBoV1, +3 aa vs. PBoV1, + 6 aa vs. RBoV and BPV) and its loop conformation advancing towards the twofold symmetry axis (Figure 5). In PBoV1 and RBoV, the shorter VR-III does not cover the entire twofold region, but their loop also advances the twofold axis and restricts the size of the depression. Additionally, a different loop confirmation of VR-IX in CnMV, PBoV1, and RBoV compared to HBoV1 and BPV near the twofold axis (Figure 4a) further causes an increase in the radial capsid surface in this region. Previously, a seven amino acid insertion of HBoV1 in VR-IX (aa 505–511, Figure 4a) was suggested to be a host determinant. None of the newly determined capsid structures possess this insertion, and therefore, this feature currently remains specific for primate bocaviruses.

In contrast to VR-III, CnMV’s VR-I is the shortest loop in comparison to the other bocaviruses (−3 aa vs. BPV, −4 aa vs. HBoV1, −6 aa vs. PBoV1 and RBoV) and is located between the threefold protrusion and the fivefold region (Figure 5). The VR-VI loop, while being only 1 aa shorter in CnMV, PBoV1, and RBoV compared to HBoV1 and BPV, is not surface-exposed in the former viruses caused by the much longer VR-V loop situated above this loop. The VR-VII loop is located at the base of the threefold protrusion in CnMV. Probably due to the different appearance of the threefold region, this loop also displays high structural heterogeneity among the bocaviruses (Figure 4a). The longest loop is found in RBoV (+2 aa vs. CnMV and HBoV1, +4 aa vs. PBoV1), with an up to 13 Å Cα-Cα distance.

To date, most bocavirus capsids have not been studied on a molecular level. However, recent studies have analyzed the impact of natural-occurring and engineered capsid variants of HBoV1 on infectivity and antigenicity due to the interest of using bocaviral vectors for gene therapy applications [48]. The previously mapped monoclonal antibodies to the HBoV capsids [43] are unlikely to bind to CnMV, PBoV1, or RBoV capsids, as the loops vary significantly in structure and amino acid sequence, as described above. However, natural exposure to these viruses may result in some seroprevalence in the human population, as the hosts of these viruses live in close proximity to humans. Consequently, a PBoV strain was isolated from an upper respiratory infection in a 3-year-old child in northeastern Iran in 2018 [25]. Similar crossings of species have been shown previously for parvoviruses. For example, the emergence of Canine parvovirus (CPV) in the 1970s was a result of cross-species transfer from Feline panleukopenia virus (FPV), resulting in a deadly pandemic in canines [49]. Thus, studying these viruses is not only of interest for the health of livestock and pets, but also a public health concern.

## 4. Conclusions

The *bocaparvovirus* genus contains a large group of virus species in the *Parvovirinae* subfamily, with members infecting a wide range of vertebrate hosts. Prior to this study, only the primate bocaviruses, HBoV1-4 and GBoV1, and bovine parvovirus, BPV, had been structurally characterized, covering only a small number of the viruses host diversity of this extensive genus. The determination of the CnMV, PBoV1, and RBoV capsid structure expands the structural atlas for this genus. For other genera, such as the *Protoparvovirus* genus, viruses infecting the same hosts have been described, such as CPV, porcine parvovirus, or H-1 parvovirus. However, no significant structural similarities, except for the capsid core, which is shared among all parvoviruses, were observed, and the amino acid sequence identities among the viruses of the same host range from 17 to 23%. 

**Figure 6:**
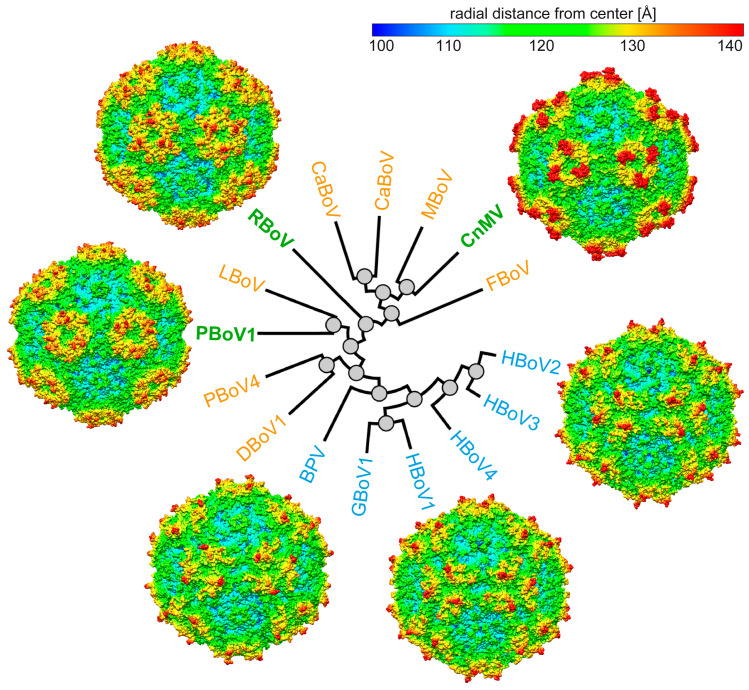
Capsid structures of the genus *Bocaparvovirus*. Radial dendrogram of selected bocaviruses using their VP2 amino acid sequence, generated at https://ngphylogeny.fr/ (accessed on 12 July 2023). The viruses for which capsid structures were previously determined are colored blue, those that are determined in this study are colored green, and the viruses without a known capsid structure are orange. Capsid surface maps based on the atomic model are shown for CnMV, HBoV2, HBoV1, BPV, PBoV1, and RBoV (ordered clockwise). The maps are radially colored (blue to red) according to distance to the capsid center, as indicated by the scale bar. DBoV: dromedary camel bocavirus, LBoV: rabbit bocavirus, MBoV: mink bocavirus, CslBoV: California sea lion bocavirus, CaBoV: canine bocavirus, FBoV: feline bocavirus.

Compared to the previously published bocavirus capsid structures, the CnMV, PBoV1, and RBoV capsids show a higher level of structural heterogeneity (Figure 6). However, currently, many of these viruses are poorly understood. Thus, the capsid structures will help future characterization of these pathogens on a molecular level, in particular, in the absence of antivirals or vaccines.

## Figures and Tables

**Table 1:** Summary of data collection, image processing, and refinement statistics.

Parameter	CnMV	PBoV1	RBoV
Total no. of micrographs	653	7949	7616
Defocus range (µm)	0.5–2.0	0.8–2.1	0.8–2.1
Electron dose (e^−^/Å^2^)	59	50	50
No. of frames/micrograph	52	50	50
Pixel size (Å/pixel)	0.92	0.95	0.95
No. of particles used for final map	50,866	98,388	9199
Resolution of final map (Å)	2.72	2.31	2.52
PHENIX model refinement statistics			
Map CC	0.84	0.88	0.88
RMSD Bonds (Å)	0.01	0.01	0.01
RMSD Angles (°)	0.83	0.90	0.82
All-atom clash score	9.34	7.81	7.95
Ramachandran plot (%)			
Favored	97.8	97.9	98.5
Allowed	2.2	2.1	1.5
Outliers	0.0	0.0	0.0
Rotamer outliers	0.0	0.0	0.0
No. of C_β_ deviations	0	0	0

## Data Availability

The CnMV, PBoV1, and RBoV cryo-EM-reconstructed density maps and models built for their capsids were deposited in the Electron Microscopy Data Bank (EMDB) with the accession numbers EMD-41614/PDB ID 8TU0 (CnMV), EMD-41615/PDB ID 8TU01 (PBoV1), and 41616/PDB ID 8TU2 (RBoV).
